# Social complexity affects cognitive abilities but not brain structure in a Poeciliid fish

**DOI:** 10.1093/beheco/arae026

**Published:** 2024-04-02

**Authors:** Zegni Triki, Tunhe Zhou, Elli Argyriou, Edson Sousa de Novais, Oriane Servant, Niclas Kolm

**Affiliations:** Behavioral Ecology Division, Institute of Ecology and Evolution, University of Bern, Baltzerstrasse 6, 3012 Bern, Switzerland; Department of Zoology, Stockholm University, Svante Arrheniusväg 18 B, 10691, Stockholm, Sweden; Brain Imaging Centre, Stockholm University, Svante Arrheniusväg 16 A, 10691, Stockholm, Sweden; Department of Zoology, Stockholm University, Svante Arrheniusväg 18 B, 10691, Stockholm, Sweden; Behavioural Ecology Laboratory, Faculty of Science, University of Neuchâtel, Emile-Argand 11, 2000 Neuchâtel, Switzerland; Department of Zoology, Stockholm University, Svante Arrheniusväg 18 B, 10691, Stockholm, Sweden; Department of Zoology, Stockholm University, Svante Arrheniusväg 18 B, 10691, Stockholm, Sweden

**Keywords:** associative learning, brain morphology, executive functions, group size, group composition, inhibitory control, reversal learning, X-ray

## Abstract

Some cognitive abilities are suggested to be the result of a complex social life, allowing individuals to achieve higher fitness through advanced strategies. However, most evidence is correlative. Here, we provide an experimental investigation of how group size and composition affect brain and cognitive development in the guppy (*Poecilia reticulata*). For 6 months, we reared sexually mature females in one of 3 social treatments: a small conspecific group of 3 guppies, a large heterospecific group of 3 guppies and 3 splash tetras (*Copella arnoldi*)—a species that co-occurs with the guppy in the wild, and a large conspecific group of 6 guppies. We then tested the guppies’ performance in self-control (inhibitory control), operant conditioning (associative learning), and cognitive flexibility (reversal learning) tasks. Using X-ray imaging, we measured their brain size and major brain regions. Larger groups of 6 individuals, both conspecific and heterospecific groups, showed better cognitive flexibility than smaller groups but no difference in self-control and operant conditioning tests. Interestingly, while social manipulation had no significant effect on brain morphology, relatively larger telencephalons were associated with better cognitive flexibility. This suggests alternative mechanisms beyond brain region size enabled greater cognitive flexibility in individuals from larger groups. Although there is no clear evidence for the impact on brain morphology, our research shows that living in larger social groups can enhance cognitive flexibility. This indicates that the social environment plays a role in the cognitive development of guppies.

## Introduction

Animals display impressive cognitive abilities, from simple associative learning (Bielecki et al. 2023) to complex and sophisticated cognitive skills, such as tool use (Finn et al. 2009), problem-solving (Damerius et al. 2017) and theory of mind (Call and Tomasello 2008). Nevertheless, there is tremendous variation in their performance. Large-scale phylogenetic comparisons have revealed patterns and generated hypotheses as to why such variation exists. They suggest that species may evolve to adjust their brain morphology to their cognitive needs based on the ecological conditions (Shultz and Dunbar 2006; van Schaik and Burkart 2011). Over decades of research into this question, the positive correlations between the brain, cognitive traits and multiple ecological conditions led to the emergence of several “intelligence” hypotheses. For instance, the “social brain hypothesis” (Dunbar 1998) states that the brain or specific brain regions have enlarged due to selective social pressures linked to factors such as group size (Dunbar 1992, 1993; Barton 1996; Kudo and Dunbar 2001; Beauchamp and Fernández-Juricic 2004; Shultz and Dunbar 2006; Street et al. 2017), mating systems (Pawłowski et al. 1998; Iwaniuk 2001; Barton 2006), and social bonds (Dunbar and Shultz 2007; Emery et al. 2007). On the other hand, the “ecological intelligence hypothesis” suggests that environmental conditions, like foraging ecology, are the best correlates of brain morphology and cognitive abilities (Clutton-Brock and Harvey 1977; Iwaniuk and Nelson 2001; Hutcheon et al. 2002; DeCasien et al. 2017; Rosati 2017). There is an ongoing debate on the relative importance of these hypotheses (Powell et al. 2017; González-Forero and Gardner 2018), primarily due to the varying and conflicting research outcomes when testing various clades and taxa with varying biology and ecology (DeCasien and Higham 2019; Kappeler 2019). Therefore, studying species of closely related species of the same clade would eliminate some of these inherent biological and ecological variables.

With their ability for continuous adult neurogenesis and neuronal regeneration (Zupanc 2008), teleost fishes are an ideal study clade for understanding the ecological pressures that drive brain and cognition evolution (Bshary and Triki 2022). Such plasticity also offers the possibility to adopt a within-species approach to investigate brain and cognitive development to complement comparative phylogenetic studies. For instance, the social brain hypothesis, originally emerging from between-species comparisons, can be used to explain how social pressures impact individual brain morphology (Kotrschal et al. 2012; Fischer et al. 2015; Triki et al. 2019) and cognitive performance (Brown and Braithwaite 2005; Ashton et al. 2018; Triki et al. 2020). This gives rise to an ontogenetic version of the social brain hypothesis, with the potential to put the social brain hypothesis to empirical testing. There is currently limited evidence of how living in a socially rich environment can shape fish brain morphology (Gonda et al. 2009; Kotrschal et al. 2012; Fischer et al. 2015; Triki et al. 2019), with a knowledge gap regarding the cognitive correlates. In addition, most research on social enrichment fails to consider other social pressures that can arise from interactions with different species (Bijl and Kolm 2016; Oliveira and Bshary 2021). Therefore, it is crucial to adopt an integrative approach with an experimental framework that simultaneously investigates brain morphology and cognitive performance to understand how fish adjust to social pressures arising not only from living in larger groups but also from social interactions within vs between species.

Here, we used the guppy (*Poecilia reticulata*) as a study system. In the wild, guppies live in shoals varying in size from only two fish to up to 50 per shoal, with frequent fission-fusion events allowing them to form complex and well-structured social networks (Croft et al. 2003, 2004). Also, guppies often coexist and may compete with other fish species over resources (Anaya-Rojas et al. 2021). In our experiment, we reared sexually mature female guppies in the same- and mixed-species groups that varied in size for 6 months. We established 3 different social treatments: (1) a small conspecific group of 3 guppies living together, (2) a large heterospecific group of 3 guppies living with 3 other female fish of a different species, the splash tetra (*Copella arnoldi*)—a species that coexist with guppies in nature (Phillip 1998), and (3) a large conspecific group of 6 guppies living together. This allowed us to simultaneously test the effects of group size and same- vs mixed-species group composition. In order to gain insights into how the social treatment may have impacted the social interactions of guppies, we recorded their behavior to determine if living in larger groups would lead to escalated conflicts.

**Fig. 1.**
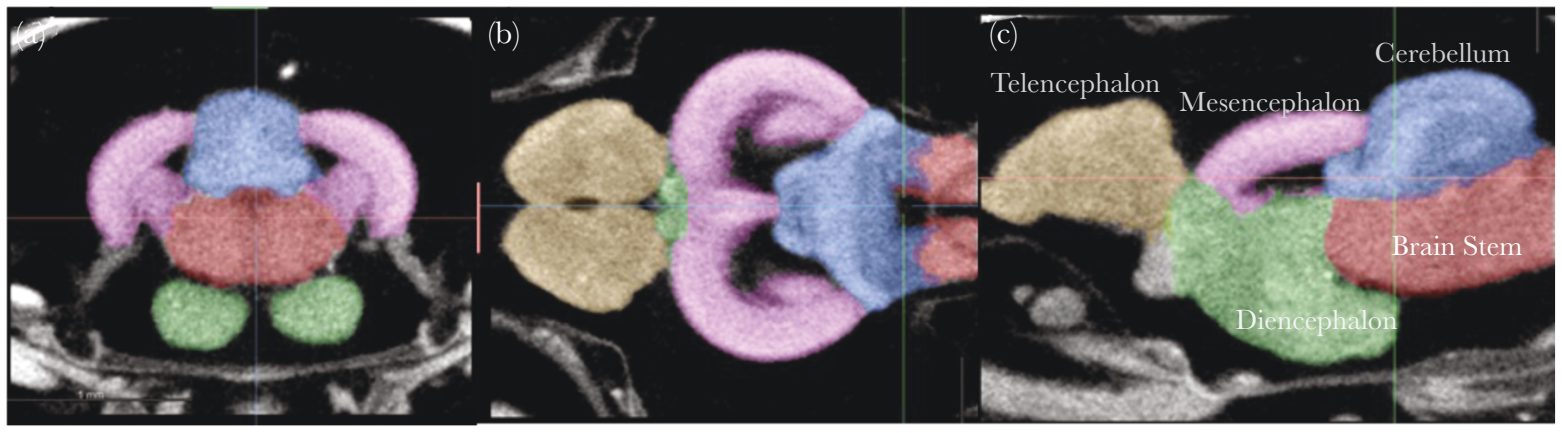
Segmented fish brain X-ray images. (a) Transversal, (b) coronal, and (c) sagittal planes of a brain scan. The major five brain parts were segmented and displayed in different colours: yellow–telencephalon, purple–mesencephalon, green–diencephalon, blue–cerebellum, and red–brain stem.

To evaluate whether the guppies’ cognitive abilities were affected by the social treatment, we chose 3 cognitive tasks: inhibitory control (cylinder test), associative learning and reversal learning. Associative learning tests basic operant conditioning abilities, while inhibitory control and reversal learning tasks test for the two executive function abilities, self-control and cognitive flexibility. These are two top-down executive functions that regulate several cognitive subprocesses and, hence, modulate complex cognition dynamics (Miyake et al. 2000; Diamond 2013). The cylinder test has been widely used to evaluate animal self-control capabilities (Kabadayi et al. 2018), from primates and birds (MacLean et al. 2014) to fish (Lucon-Xiccato et al. 2017; Triki et al. 2023a; Guadagno and Triki 2024). It consists of placing a food reward inside a transparent cylinder. The performance is then evaluated by recording whether an animal would delay their gratification and move around the cylinder without touching it—an indicator of inhibitory control ability—or whether they would bump into the cylinder in an attempt to retrieve the food immediately, which indicates a lack of inhibitory control (Kabadayi et al. 2018). In the associative and reversal learning tests, researchers in the field of animal cognition often employ the 2-color discrimination paradigm. The test evaluates the animal’s abilities in associating a color cue with a food reward. Once this association is formed, the test then reverses the color-reward contingency (reversal learning), and it allows us to estimate the animal performance by unlearning the previous rule and updating it with the new color-reward association. Such capacity to update a learning rule is an indicator of possessing cognitive flexibility abilities (Uddin 2021). Furthermore, to investigate whether there is a link between brain morphology and cognitive performance in the tested fish, we used X-ray imaging technology (see Methods) to generate fine-tuned and high-quality volume data of the major fish brain regions (White and Brown 2015). These regions included the telencephalon, diencephalon, mesencephalon, cerebellum, and brain stem (Fig. 1).

Our choice of the cognitive tests builds on the comparative research indicating that species living in larger groups tend to have larger brains (Dunbar and Shultz 2007), where larger brains and specific brain regions exhibit greater abilities in inhibitory control and cognitive flexibility (Deaner et al. 2007; MacLean et al. 2014). It also builds on Ashton et al.‘s work (2018) on magpie birds, revealing a positive correlation between group size and performance in the 3 cognitive tests: inhibitory control, associative learning, and reversal learning. Our experiment aimed to test whether the social brain hypothesis applies to individual development over an ontogenetic timescale within an experimental framework. We predict that living in a larger group will have a positive impact on cognitive performance in tasks related to inhibitory control and reversal learning. However, we do not predict to see the same effect in associative learning, since research suggests that forming associations does not necessarily require a complex neural system (Bielecki et al. 2023). The expected positive relationship between group size and performance in inhibitory control and reversal learning will be facilitated by rapid changes in brain morphology, specifically, the enlargement of certain brain regions.

## Materials and methods

### Study animals and experimental set-up

We conducted the study between December 2019 and January 2021 in the fish laboratory facilities at Stockholm University in Sweden. Our study animals were laboratory-bred guppies *Poecilia reticulata,* descendants from an initial population of more than 500 fish caught in 1998 from Quare River in Trinidad. To create a new generation of naïve guppies, we set up 75 breeding pairs in separate 2 L tanks. We regularly isolated the fry and housed them in 2 L tanks with a maximum capacity of 6 per tank. We periodically removed the ones that developed into males by displaying secondary sexual traits like color patterns, housing them in separate tanks, and ensuring that the remaining female numbers were adjusted to 6 per tank. On average, guppies reach sexual maturation within 3 months of age. We also used another fish species, the splash tetra, *Copella arnoldi*, an introduced species that is widespread across the native Trinidadian rivers where the guppy typically resides (Kenny 2008). We obtained the splash tetras from an aquarium fish supplier in Stockholm. Finally, we used 144 female guppies from our new generation and 36 female splash tetras (divided into two batches) to create 3 social treatments with different fish densities and compositions.

The 3 social treatments were: (1) a small conspecific group of 3 female guppies, (2) a large heterospecific group of 3 female guppies and 3 female splash tetras, and (3) a large conspecific group of 6 female guppies. We used only females to avoid mating and reproduction occurring and potential male–male aggression. Every treatment had 12 replicate tanks, but some replicates had a later development of apparent males whose color patterns had not yet developed when we established the treatments at the age of about 3 months. This led to eliminating one replicate in the treatment of the small conspecific group, one in the large heterospecific group, and 4 in the large conspecific group. After that, the sample size was 33 fish (11 tank replicates) in the small conspecific group, 33 fish (11 tank replicates) in the large heterospecific group, and 48 fish (8 tank replicates) in the large conspecific group treatments. All housing tanks were of 6 L capacity and contained identical enrichment of 2 cm of gravel, one plastic plant in the middle and an air filter (see Supplementary Material). We ensured an ambient temperature of ~26 °C and a light:dark cycle of 12:12 h with an ad libitum feeding schedule alternating between fish flakes and live *Artemia* (brine shrimp) hatchlings 6 days per week. Furthermore, we conducted behavioral observations on the social and foraging interactions of guppies in 17 of the tanks, finding limited evidence of behavioral variation between the social treatments (see Supplementary Material).

After 6 months, we terminated the 3 social treatments and allocated our focal individuals, i.e. the female guppies, to “baseline” and “test” sets while transferring the splash tetras to a 200 L housing tank. The baseline set served to test for brain morphology changes right after the termination of the social treatment but before the cognitive tests. In contrast, we subjected the test set first to a battery of cognitive tasks, which lasted about 50 days, and then measured their brains (see below). In the baseline set, there were ten fish from the small conspecific group, ten from the large heterospecific group, and 9 from the large conspecific group treatments. The remaining fish (23 from the small conspecific group, 23 from the large heterospecific group, and 39 from the large conspecific group treatments, see Supplementary Material) were housed individually in experimental aquaria (L × W × H; 40 × 15 × 15 cm). To avoid potential observer bias, the test fish had running number labels (1, 2, 3, etc.) to conceal their social treatment identity throughout the experiment. Each experimental aquarium had an identical enrichment of 2 cm of gravel and an artificial plant and had continuously aerated water; it also had two adjacent guillotine doors, one see-through and one opaque, dividing the space into a housing compartment and a test compartment. The experimental room had an ambient temperature of ~26 °C with a light schedule of 12 h light and 12 h dark. We fed the guppies ad libitum with defrosted adult brine shrimps delivered with a 1 mL transparent plastic pipette 6 days per week. This facilitated acclimating the fish to receive food from plastic pipettes, later used to provide food as a positive reinforcement in the learning tests. During the weekdays, when we ran cognitive tasks, fish acquired food solely from test trials. The tests started after an acclimation period of 5 days, with no trials on the weekends. During the tests, the between-trial interval for every fish was about 60 min, and one test trial per fish took about 1 min. Furthermore, there was always at least one day break between every two cognitive tests. Unfortunately, 5 fish out of the total 85 died during the experiment after jumping out of the experimental tanks during the night. That left 22 fish from the small conspecific group treatment, 21 fish from the large heterospecific group treatment, and 37 fish from the large conspecific group treatment.

### Cognitive tests

#### Inhibitory control task (detour task)

After the acclimation period, we trained fish to associate the color green with a food reward. To do so, we placed a green disc in the test compartment and delivered a defrosted adult artemia placed right on top of the disc. We repeated this exposure twice a day for 4 consecutive days. On the following day, we introduced a transparent Plexiglas cylinder (5 cm in length and 4 cm ⌀) open on either side in the test compartment. The cylinder contained a food reward placed on top of a green spot drawn inside the cylinder. This was a one-time acclimation opportunity for the fish to explore the transparent barrier. After that, fish received 3 trials on test Day 1, 3 trials on Day 2, and 4 trials on Day 3. A trial started when the experimenter simultaneously pulled up the opaque and transparent guillotine doors and allowed the fish to detour the physical barrier, here as the cylinder walls, and swim inside the cylinder to retrieve the food reward. The experimenter recorded whether the fish touched the cylinder (failure) or not (success) before retrieving the food (see Supplementary Videos). Finally, we ranked the proportion of correct detours (the number of successes divided by test trials) in descending order (80 to 1), where the largest proportion was ranked 80, reflecting thus the highest performance in our fish given that the sample size was 80 fish.

#### Associative and reversal learning tasks

In the associative learning task, we exposed the fish to a 2-color choice test to estimate fish learning abilities through operant conditioning in associating a food reward with a color cue, yellow vs red. These colors were chosen based on the guppies’ ability to detect them (Archer et al. 1987). In the test compartment, we placed two 1 mL plastic pipettes covered with either yellow or red adhesive tape, and each contained a defrosted adult artemia. An opaque gray Plexiglas rectangle plate separated the two pipettes, thus creating two zones of choice. A test trial started with the experimenter pulling up the opaque sliding door followed by the see-through door, giving the fish a few seconds to see the set-up before entering the test compartment and choosing one of the two pipettes. The experimenter scored a choice as “correct” if a fish entered the zone of the rewarding color with its body length at its first attempt and “failure” if it chose the non-rewarding color at its first attempt (see Supplementary Videos). We balanced the color and side of the rewarding pipette across fish and trials. As such, half of the fish had red as the rewarding color while the other half had yellow with 50–50 presentation of the rewarding color on the left and right side of the test compartments (with no more than 3 presentations on the same side in succession) (following the protocol by Triki et al. (2022b) for guppies).

Once a fish learned the color-reward association in the associative learning phase, we tested its abilities in a reversal learning phase. It consisted of reversing the reward contingency, and the previously unrewarding color became the new rewarding cue. For example, if a fish learned the yellow-reward association in the previous task, in the reversal task, it had to learn the red-reward association instead. For associative and reversal learning phases, the fish received twenty test sessions, with one session per day (one session = 6 trials). We set the learning criteria in each test to a score of either 6 correct choices out of 6 consecutive trials or 5 correct choices out of 6 trials in two consecutive sessions. The probability of learning by chance with these criteria is *P* < 0.05 (with a binomial test).

Finally, for the associative learning performance, we ranked fish success and the number of sessions needed to learn the task in descending order (from 80 to 1), where the smallest number of sessions to succeed was ranked as 80, reflecting thus the highest performance in our fish. In the reversal learning performance, we ranked fish success and the number of sessions needed to pass first the associative learning phase and then the reversal phase in descending order (from 80 to 1), where the smallest number of sessions to succeed was ranked as 80, reflecting thus the highest performance in our fish given that the sample size was 80 fish.

### Brain staining and 3D-image acquisition

We prepared the 29 female guppies from the baseline group and 80 from the treatment group for X-ray brain scans by first euthanizing them with an overdose of benzocaine (0.4 g L^−1^). With a digital caliper, we estimated the fish body size as standard length (SL) to the nearest 0.01 millimeter. We then fixated their whole heads in 4% paraformaldehyde phosphate-buffered saline (PBS) for 5 days. After that, we washed the samples twice in PBS for 10 min and kept them in PBS. We then followed the Phosphotungstic acid (PTA) staining protocol by Lesciotto et al. (2020) to prepare our samples for X-ray scanning. In this protocol, we first dehydrated the samples by placing them in a series of solutions as follows: one day in 30% ethanol in PBS; one day in 50% ethanol in PBS; one day in 70% ethanol in PBS; one hour in a solution with a ratio of 4:4:3 volumes of ethanol, methanol, and H2O; one hour in 80% methanol in H2O; and 1 h in 90% methanol in H2O. After that, we proceeded with the staining phase by placing the samples in 0.7% PTA in 90% methanol in H2O for 23 days. Twenty-three days was the optimal staining duration for our samples based on pilot rapid X-ray scans to check the staining quality (see below). We then rehydrated the samples by placing them in 90% methanol for 6 h; 80% methanol overnight; 70% methanol for 6 h; 50% methanol overnight; 30% methanol for a day; in PBS for one day; and finally storage in 0.01% sodium azide in PBS.

We transferred the samples to the Stockholm University Brain Imagery Center (SUBIC) for image acquisition. We scanned the samples using a Zeiss Xradia Versa 520, with the X-ray source at a voltage of 100 kV and a power of 9W. We used the 0.4× objective coupled with a scintillator. The source-to-sample distance was 30 mm, and the sample-to-detector distance was 81 mm. The effective voxel size was 9.17 μm with a compensated optical and geometrical magnification. The scan consisted of 1201 projections over 360 degrees with 1 s exposure time for each projection. Each scan took 1 h and 36 min on average, including reference images and the readout time of the CCD camera. Given the small size of fish heads and to optimize the scan time, we arranged 4 samples per scan (see Supplementary Material). We obtained 3-dimensional images of the brain scans through an automatized tomography reconstruction with Zeiss Scout-and-Scan software.

### Brain morphology measurements

To segment the 3D brain images, we first aligned the images digitally in Dragonfly (Dragonfly 2020.2 [Computer software] 2020) in 3 planes—transversal, coronal, and sagittal to the cardinal axes (Fig. 1). To ensure accuracy, we either adjusted the voxel size of the dataset through resampling using bicubic interpolation or made note of any changes in voxel size and corrected the volume accordingly. This was necessary due to the potential for minor changes in voxel sizes caused by alignment. We obtained full head images in the scans and then cropped them to include only the brain tissue. This was done consistently across all planes to improve segmentation and accurately measure the volumes.

For the segmentation *per* se, we first generated a semi-manually segmented brain into 5 regions (telencephalon, mesencephalon, diencephalon, cerebellum, and brain stem) (Fig. 1). This was achieved by employing Biomedisa (Lösel et al. 2020) using random walker interpolation between sparsely manually segmented slices. In total, we semi-manually segmented 23 samples and used them as an Elastix template (Klein et al. 2009). We then manually checked these samples and corrected potential errors, then used them as a training dataset for the following deep-learning-based segmentation on the rest of the samples. We used the deep-learning algorithm from Schoppe et al. (2020), which is U-net-like (Ronneberger et al. 2015). Finally, we computed the brain regions’ volumes by multiplying the voxel number and voxel size from the segmented labels.

### Data analysis

We used the open-access software R, version 4.2.1 (R Core Team 2022), to run all statistical analyses and generate the figures. Overall, we implemented 4 different statistical analysis approaches.

First, we tested whether the fish exposed to different social treatments may have developed different cognitive abilities. We analyzed the rank performance data for all 3 abilities: inhibitory control, associative learning, and reversal learning. Given that this data violates the overdispersion assumption for count data, we fitted 3 generalized linear mixed models using a template model builder (glmmTMB) with a negative binomial distribution. In one model, we fitted inhibitory control rank performance as the dependent variable, social treatment as the predictor and batch as a random factor. For the associative and reversal learning models, we fitted rank performance as the dependent variable, treatment as a predictor, and the color of the pipette as a covariate to control statistically for potential color bias, while the batch was the random factor.

Second, we tested whether brain morphology (total brain size and brain region sizes) differed across the 3 social treatments in both the baseline and test groups. To do so, we ran 2 linear mixed effect models (LMMs), one for baseline data and one for test data, where we fitted log-transformed brain size (mm^3^) as the dependent variable, social treatment (with 3 levels: small conspecific group, large heterospecific group, and large conspecific group) as the fixed predictor, log-transformed and standardized body size (mm) as a covariate, and batch number (we had 2 batches, see above) as a random factor. Similarly, for the 5 brain regions, telencephalon, diencephalon, mesencephalon, cerebellum, and brain stem, we fitted 2 multivariate analyses of variance (MANOVA) for baseline and test data. We fitted the dependent variable as a matrix of all 5 brain region sizes log-transformed and standardized (e.g. Triki et al. 2019), with treatment as a predictor and body size as a covariate. We also fitted batch as a predictor since MANOVA does not support mixed effects.

Third, we tested whether cognitive performance was influenced by individual brain size and region size. In preparation for these analyses, we extracted the residuals of log brain size on log body size, as well as the residuals of every brain region size (log-transformed) on log-transformed and standardized brain and body sizes. Then, we fitted a set of glmmTMB models with the designated cognitive ability as the dependent variable and social treatment and the designated brain measurement as predictors. We also fitted batch as the random factor in all these models, while for the models testing for associative and reversal learning, we added the color of the pipette as a covariate.

Finally, we checked that the fitted models met their corresponding assumptions, such as normality of residuals and homogeneity of variance. For further details, we provide a step-by-step code and data used to generate the findings in the present study (see the Data and Code accessibility statement).

## Results

### Social treatment effect on cognitive performance

**Fig. 2.**
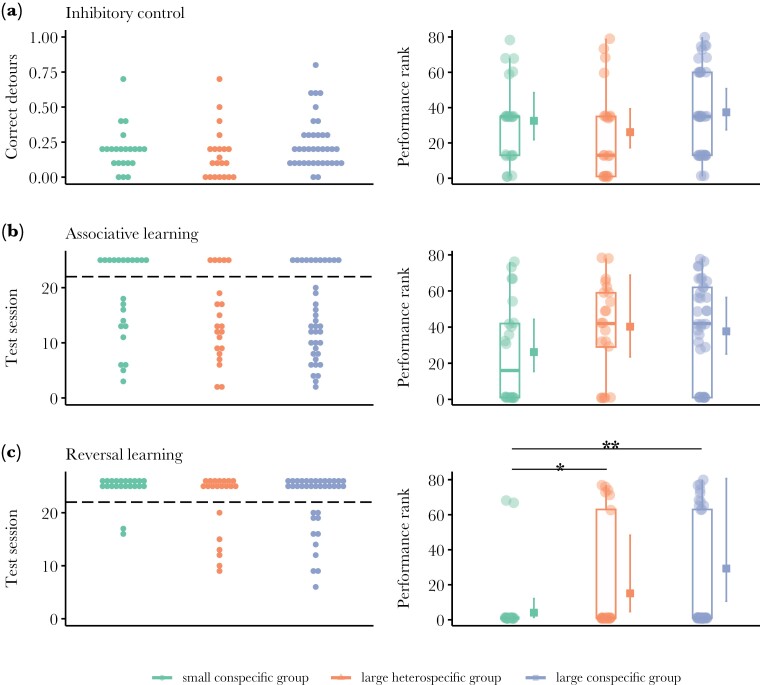
Cognitive performance of the guppies from the 3 social treatments. The panels on the left show the raw scores per cognitive task as dot plots for inhibitory control, associative, and reversal learning. Data points above the dashed line in associative and reversal learning refer to individual guppies that failed to reach the learning criterion within 120 test trials (20 test sessions where 1 session = 6 trials). Panels on the right show the estimate and 95% CI of model marginal effects, combined with boxplots of median and interquartile of performance rank (*N* = 80) for (a) inhibitory control, (b) associative learning, and (c) reversal learning. The highest ranks refer to the highest performance. The reversal learning test shows an effect of social treatment on fish performance (*P < 0.05, **P < 0.01), but not the other 2 tests (*P* > 0.05).

**Fig. 3.**
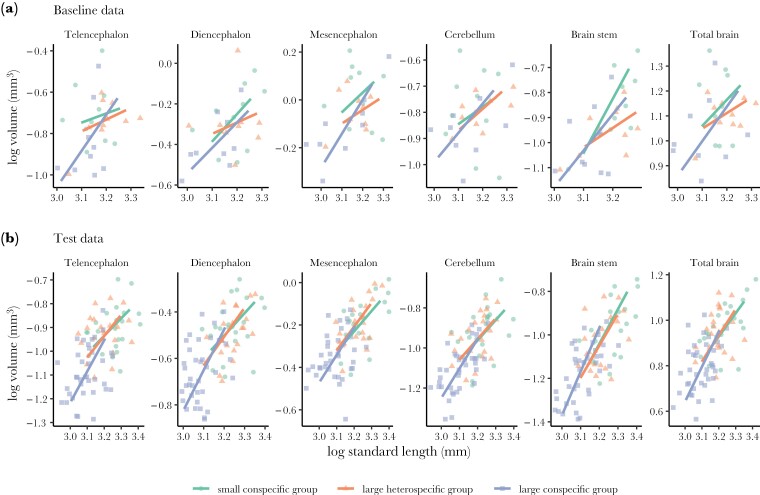
Brain morphology of the guppies from the 3 social treatments. Scatter plot and regression lines of log-normal transformed volume (mm^3^) of the brain measurement on the log-normal transformed body size (standard length in mm) as a function of social treatment from (a) the baseline dataset of the 29 female guppies sampled before the cognitive tests and (b) test dataset (80 female guppies) after the cognitive tests. There were no significant effects of social treatment on brain morphology (*P* > 0.05).

Among the 3 cognitive tests we ran, reversal learning emerged as the only one being affected by the social treatment (glmmTMB: *N* = 80, χ^2^ = 12.852, *P* = 0.002, explained variance: marginal-*R*^2^ = 0.35, conditional-*R*^2^ = 0.46). Fish from the large conspecific group and the large heterospecific group outperformed fish from the small conspecific group (posthoc test emmeans: large conspecific group vs small conspecific group, estimate = 1.971, *P* = 0.004; large heterospecific group vs small conspecific group, estimate = 1.309, *P* = 0.047), with no statistically significant differences between the large conspecific group and the large heterospecific group (estimate = 0.662, *P* = 0.584) (Fig. 3). The other 2 tests, performance in the inhibitory control, and associative learning tasks did not significantly differ across the 3 social treatments (glmmTMB: inhibitory control, *N* = 80, χ^2^ = 1.952, *P* = 0.376; associative learning, *N* = 80, χ^2^ = 1.611, *P* = 0.446) (Fig. 2).

### Social treatment effect on brain morphology

Guppies’ brain morphology did not change as a function of social treatment. Neither fish sampled before (*N* = 29) nor those sampled after (*N* = 80) the cognitive tests showed a significant (*P *> 0.05) change in overall brain size or the 5 regions quantified here (telencephalon, diencephalon, mesencephalon, cerebellum, and brain stem) (Fig. 3) (see detailed statistics in Table 1). All measurements were corrected for body size since body growth across treatments varied significantly. Guppies from the large conspecific group were significantly smaller than guppies in both the small conspecific group and the large heterospecific group (LMM: from baseline data: χ^2^ = 7.843, *P* = 0.019, *R*^2^ = 0.22; from test data: χ^2^ = 129.5, *P* < 0.001, marginal-*R*^2^ = 0.55; conditional-*R*^2^ = 0.66, see Supplementary Fig. S4).

**Table 1.** Summary table of the outcomes of brain morphology as a function of social treatment.

Dataset	Explanatory variable	test	*N*	*F*-value	*P-*value
*Total brain size*					
Baseline	Social treatment	LMM	29	1.458	0.482
	Body size			**13.421**	**<0.001**
Test	Social treatment	LMM	80	0.359	0.835
	Body size			**59.764**	**<0.001**
*Telencephalon, diencephalon, mesencephalon, cerebellum, and brain stem sizes*					
Baseline	Social treatment	MANOVA	29	0.362	0.955
	Body size			2.542	0.065
	Batch identity			1.920	0.140
	Social treatment × batch identity			0.389	0.943
Test	Social treatment	MANOVA	80	0.908	0.527
	Body size			**14.598**	**<0.001**
	Batch identity			**2.344**	**0.050**
	Social treatment × batch identity			0.499	0.887

Values in bold refer to statistically significant results with a *P*-value ≤ 0.05.

### The link between cognitive performance and brain morphology as a function of social treatment

**Fig. 4.**
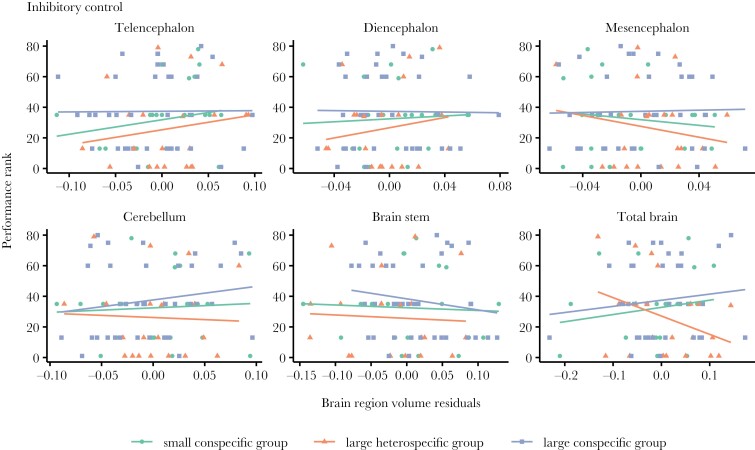
The relationship of inhibitory control performance and brain morphology of the guppies from the 3 social treatments. Scatter plot and regression lines of performance rank (where highest ranks refer to highest performance) on brain measurement residuals (x-axes) (*N* = 80). No significant effect was detected (*P* > 0.05).

**Fig. 5.**
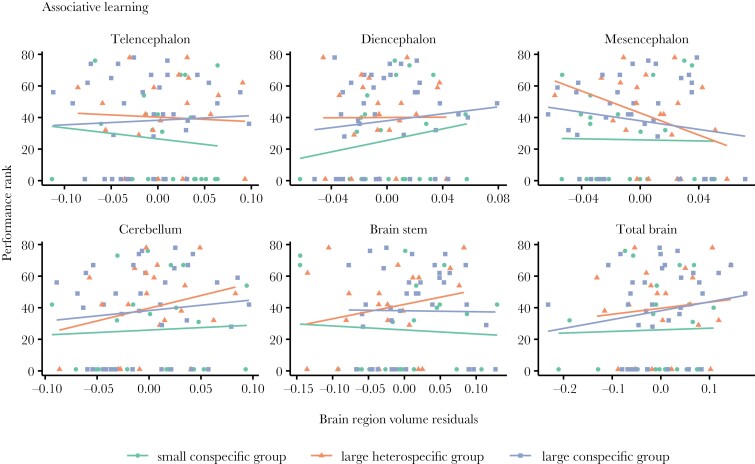
The relationship of associative learning performance and brain morphology of the guppies from the 3 social treatments. Scatter plot and regression lines of performance rank (where highest ranks refer to highest performance) on brain measurement residuals (x-axes) (*N* = 80). No significant effect was detected (*P* > 0.05).

**Fig. 6.**
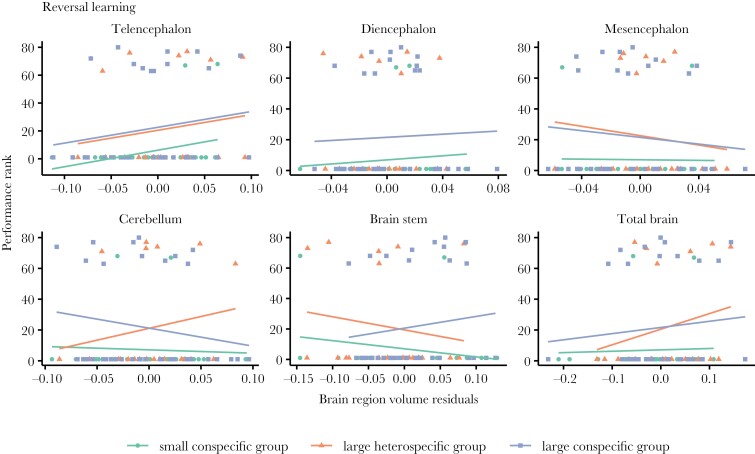
The relationship of reversal learning performance and brain morphology of the guppies from the 3 social treatments. Scatter plot and regression lines of performance rank (where highest ranks refer to highest performance) on brain measurement residuals (x-axes) (*N* = 80). Only the telencephalon relative size correlated significantly with performance (*P < 0.05).

By looking at individual performance in every test (Figs. 4–6) and correlating it to brain morphology and social treatment, we found that relative telencephalon size was positively associated with improved performance in the reversal learning task (glmmTMB: *N* = 80, telencephalon size residuals, χ^2^ = 6.374, *P* = 0.011, marginal-*R*^2^ = 0.45, conditional-*R*^2^ = 0.55, Fig. 6), but independently of social treatment (glmmTMB: *N* = 80, telencephalon size residuals × social treatment, χ^2^ = 1.319, *P* = 0.517) (Fig. 4). For the other brain measurements as well as the other 2 cognitive tasks, i.e. inhibitory control and associative learning, we did not find any statistically significant relationships (Fig. 4, see detailed statistics in Table 2).

**Table 2.** Summary table of the outcomes of cognitive performance as a function of brain morphology and social treatment.

	Inhibitory control			Associative learning			Reversal learning		
Explanatory variable	χ^2^	*P*	marginal *R*^2^/ conditional *R*^2^	χ^2^	*P*	marginal *R*^2^/ conditional *R*^2^	χ^2^	*P*	marginal *R*^2^/ conditional *R*^2^
Telencephalon	0.665	0.415	0.054/ NA	0.034	0.852	0.056/ NA	**6.374**	**0.011**	**0.45/ 0.55**
Social treatment	2.449	0.294		1.691	0.429		**19.509**	**< 0.001**	
Telencephalon × Social treatment	0.505	0.777		0.450	0.798		1.319	0.517	
Diencephalon	0.157	0.692	0.043/ NA	0.793	0.373	0.113/ 0.151	2.392	0.121	**0.40/ 0.55**
Social treatment	2.010	0.366		2.183	0.336		**16.342**	**< 0.001**	
Diencephalon × Social treatment	0.376	0.828		0.736	0.692		0.132	0.936	
Mesencephalon	0.299	0.584	0.050/ NA	0.933	0.334	0.051/ NA	3.790	0.051	**0.42/ 0.64**
Social treatment	1.934	0.380		1.861	0.394		**18.759**	**< 0.001**	
Mesencephalon × Social treatment	0.600	0.741		0.429	0.807		3.421	0.181	
Cerebellum	0.249	0.618	0.042/ NA	0.575	0.448	0.042/ NA	1.231	0.267	**0.39/ 0.61**
Social treatment	1.969	0.374		1.635	0.442		**15.436**	**< 0.001**	
Cerebellum × Social treatment	0.305	0.859		0.114	0.944		3.521	0.172	
Brain stem	0.474	0.491	0.043/ NA	0.010	0.919	0.045/ NA	0.034	0.853	**0.39/ 0.56**
Social treatment	2.278	0.320		1.508	0.471		**15.056**	**< 0.001**	
Brain stem × Social treatment	0.134	0.935		0.190	0.909		4.688	0.096	
Total brain	0.033	0.856	0.100/ NA	0.292	0.588	0.044/ 0.049	2.387	0.122	**0.38/ 0.55**
Social treatment	2.610	0.271		1.476	0.478		**15.042**	**< 0.001**	
Total brain × Social treatment	3.711	0.156		0.094	0.954		0.922	0.631	

Values in bold refer to statistically significant results with a *P*-value ≤ 0.05. The sample size is *N* = 80 guppies. NA: not applicable.

## Discussion

Our study tested the social brain hypothesis within an ontogenetic timescale. We asked whether social group size and group composition affect brain morphology and cognitive performance across different cognitive domains in a guppy. The key findings were: (1) living in a large group of 6 individuals, either of conspecifics or heterospecifics, yielded improved performance in the reversal learning task than those in the small conspecific group of 3 individuals; (2) social treatment did not affect associative learning performance and inhibitory control; (3) social treatment did not affect brain morphology; and (4) independently of social treatment, relative telencephalon size correlated positively with individual performance in the reversal learning task. We discuss each finding in turn in the following paragraphs.

Living in larger groups can create richer social environments and lead to the development of more sophisticated strategies to cope with daily challenges (Dunbar and Shultz 2007). This, in turn, can result in more advanced cognitive abilities. Our study found that guppies living in larger groups of 6 individuals exhibited better cognitive flexibility, as expected based on the social brain hypothesis. This aligns with previous studies finding a positive correlation between social complexity and cognitive flexibility (Byrne and Whiten 1989; Bond et al. 2007; Ashton et al. 2018). In larger groups, individuals must be able to switch attention and adjust behaviors with changing demands more effectively than when cohabiting with fewer individuals. Interestingly, our study found that regardless of whether a guppy lived in larger groups of conspecifics or heterospecifics, their cognitive capacities developed similarly, a finding that is comparable to that by Fischer et al. (2021) on cichlids. It seems that the amount of social interactions that occur when living in larger groups is more important for cognitive flexibility than the exact nature of these interactions. Alternatively, the differences in social interactions between the two species included here might not be large enough to generate differences in the cognitive tasks we quantified. Indeed, our analysis of specific behaviors, specifically aggression and aggregation, did not provide any clear explanations for the differences observed in group performance during the reversal learning task. This result suggests that a more comprehensive ecological framework, encompassing social interactions between different species, may be necessary to comprehend the effect of the social environment on cognitive development. It would be highly interesting to extend this finding and incorporate between-species social interactions in the comparative phylogenetic analyses looking into cognitive evolution. Beyond predator–prey interactions (Bijl and Kolm 2016), this has not been done yet. Moreover, an important applied outcome of our finding is that social enrichment could be highly effective when created through the inclusion of other species, for instance, in zoos and sanctuaries (Dorman and Bourne 2010).

Unsurprisingly, there were no differences in associative learning performance due to social treatment. The test assesses basic operant conditioning abilities, and simple cognitive processes are sufficient for forming associations (Savage 1980; Bielecki et al. 2023; Triki et al. 2023a). The task is often excluded when researchers look into complex cognitive abilities, like the “general intelligence” factor (Damerius et al. 2017; Aellen et al. 2022). In sum, social complexity would unlikely impact how an individual forms basic associations, such as between a color cue and a food reward.

The ability to pause and override motor impulses in response to a specific stimulus is known as inhibitory control. When executed correctly, this results in adaptive, goal-oriented behaviors requiring complex cognitive processes (Diamond 2013). We used the detour task to test fish performance in this cognitive capacity, and the results showed that social treatment did not affect their inhibitory control abilities. This finding goes against our original predictions as previous studies have found that living in complex social environments correlates positively with enhanced inhibitory control abilities for individuals and species (Amici et al. 2008; Ashton et al. 2018; Johnson-Ulrich and Holekamp 2020). However, it is worth noting that other ecological factors, besides social complexity, may also have significant impacts on self-control (see review by Rosati (Rosati 2017)). Currently, we rely heavily on comparative and correlative research to study the relationship between social complexity and inhibitory control. Therefore, we need more experimental data at both the species and population levels to draw convincing conclusions about whether social complexity directly enhances inhibitory control capabilities.

Regarding the brain morphology analysis, there were no evident changes caused by the social treatment. Still, we noticed that the brain allometry was different, with fish from the treatment of 6 guppies having relatively steeper allometry slopes for overall brain size but also for most of the brain regions on body size, compared to the other 2 treatments (Fig. 2). It is clear that differences in body growth drove this (see Supplementary Fig. S4). Although we fed all fish ad libitum and we expected that their body growth would be density-dependent (Lorenzen and Enberg 2002), there were differences across treatments. Guppies in the small conspecific group and the large heterospecific group were of similar body sizes, but they were substantially larger than those in the large conspecific group. It suggests that guppies were more successful foragers than splash tetras in the large heterospecific group because they attained larger body sizes as if they were alone in the tank compared to those growing relatively smaller when they were competing against their peers in the large conspecific groups. Despite these body growth differences across treatments, there were no evident size changes in the brain or the 5 major brain regions when corrected for body size. We can only speculate on why we did not find any effects when other studies on fish have found substantial differences in brain morphology associated with variation in the social environment. For instance, cleaner fish living at higher population densities possess larger forebrains (telencephalon and diencephalon) (Triki et al. 2019), while 9-spined sticklebacks reared in groups develop a larger optic tectum and a smaller olfactory bulb than those reared individually (Gonda et al. 2009) (see review by Gonda et al. (2011) for more examples and detailed discussion). It is possible that we did not see any changes across the social treatments because increasing the group size from 3 to 6 was not enough to create the necessary social effects that lead to brain morphology changes. Yet, it is also possible that the social treatment generated effects on a different scale that could not be detected with our X-ray scan methods. For instance, it could be that changes in neural connectivity, neuronal activity or gene expression, while not essentially leading to volume changes, were responsible for the observed group performance differences (Weitekamp and Hofmann 2014; Herculano-Houzel 2017; Wallace and Hofmann 2021). Another possible reason for the lack of social treatment effect on brain morphology in our data is that we only manipulated group size, ignoring other key factors that exist in the wild and affect brain development, such as predation pressure, mating strategies, and feeding ecology (Brown and Braithwaite 2005; Kolm et al. 2009; Powell et al. 2017).

We found that relative telencephalon size explained, to some extent, fish performance in the reversal learning test with no apparent differences in this brain region volume across social treatments. Specifically, relative telencephalon size correlated positively with reversal learning performance within each social treatment (see Fig. 4c), but it did not explain performance differences across treatments. Individual-level improvement in reversal learning performance due to larger telencephalon has already been demonstrated in guppies by Triki et al. (2022b, 2023b). Additionally, Triki et al. (2022a, 2023b) found a positive correlation between the size of this brain part and inhibitory control abilities, which was not observed in the current study. One possible reason for this could be that Triki et al.’s studies (2022a, 2023b) involved the use of guppies that were selectively bred to reach a divergence in relative telencephalon size over several generations. Often, it is difficult to detect brain morphology effects on behavior in wild-type strains of laboratory-held animals fed ad libitum and where predation selection pressures are removed (see discussion on this topic in (McNeil et al. 2021)). Hence, while not general across all cognitive abilities assayed here, we find it interesting that the effect of relative telencephalon size on cognitive flexibility is consistent both for wild-type guppies and for artificial selection line guppies targeted for telencephalon size.

In summary, our research suggests that social complexity affects cognitive flexibility but not inhibitory control or basic operant conditioning skills. This impact was likely driven by mechanisms beyond plastic changes in the 5 major brain regions. Although there may not be any apparent changes in brain morphology, the findings suggest that living in larger social groups can affect an individual’s cognitive development, specifically their cognitive flexibility. However, further research, using experimental methods, is necessary to fully understand how social and environmental factors shape cognitive development.

## Supplementary Material

arae026_suppl_Supplementary_Material

arae026_suppl_Supplementary_Videos

## Data Availability

Analyses reported in this article can be reproduced using the data provided by Triki et al. (2024).
