# Supplementary material for: Social complexity affects cognitive abilities but not brain structure in a Poeciliid fish
Source: Behav Ecol. 2024 Apr 2;35(3):arae026. doi: 10.1093/beheco/arae026 (PMC11025466; doi:10.1093/beheco/arae026)
Supplement: arae026_suppl_Supplementary_Material [file arae026_suppl_supplementary_material.pdf]

Electronic supplementary material for: Social complexity affects cognitive abilities but not brain structure in a Poecilid fish

Supplementary findings

2-Behavioural observations

We attempted to acquire behavioural observations to establish a basic understanding of the social interactions occurring in these treatments. Within our logistic capacities, we recorded fish behaviour in 17 tanks from the second batch 5 months after the start of the treatment. In total, we recorded six tanks from the small conspecific group, six from the large heterospecific group, and five from the large conspecific group tanks. We recorded fish for 20 minutes in the morning before feeding, then provided them with food and recorded their behaviour for another 10 minutes. This resulted in behavioural observations before and after feeding. We extracted the number of potential chasings (aggression) from these videos and created image sequences from each recording with a timelapse of 2 min. The experimenter would run the video recording, and they would take a screenshot every 2 min. They then analysed these images in the open-source ImageJ software (version 1.53a), which helped estimate the distance (in cm) between fish. Such measurements served as a simplified indicator of aggregation and space used by the fish in the three social treatments. Together, such an approach helped to acquire simple measurements of fish's natural behaviour in the three treatments without disturbance.

2.1. Statistics

As explorative analyses, we looked at whether guppies behaved differently during the social treatment. We ran a set of linear mixed effects models (LMMs) to look into the aggression rates and aggregation among the fish. First, we fitted a model with aggression rates among the fish as the response variable (log-transformed), and treatment (three guppies, three guppies plus three splash tetra, and six guppies) and feeding time (before vs after feeding) as fixed predictors, and holding tank identity as a random factor. Second, we also examined whether the splash tetra attacked each other by fitting an LMM with aggression among tetras as the dependent variable, feeding time as a fixed predictor, and tank identity as the random factor. Third, we fitted an LMM with average distance among guppies as the response variable (log-transformed) and treatment and feeding time as fixed predictors, with tank identity as a random factor. Finally, we fitted another LMM with average distance among all fishes (splash tetra included) with similar predictors and random factors as the previous model. Finally, we checked that all LMMs met the assumptions of normality of the residuals' distribution and homogeneity of the variance via statistical models and visual plots.

2.2. Results

The statistical outcomes of the behavioural analyses are in Table S1. The social treatment significantly affected the aggression rates towards guppy fish (LMM: X2 = 6.198, p = 0.045,

marginal-R2 = 0.18, conditional-R2 = 0.20, Fig. S5, Table S1), apparently driven by the large heterospecific group with higher aggression rates. We did not find a statistically significant effect by looking more closely at this particular treatment and exploring whether the splash tetras show more aggression rates as a function of feeding. Still, they tend to be more aggressive when food is around than when there is no food (Fig. S6, Table S19). The statistical analyses on the distance among guppies with and without the splash tetras showed no evident effect of social treatment ($p > 0.05$, Fig. S7, Fig. S8, Table S1).

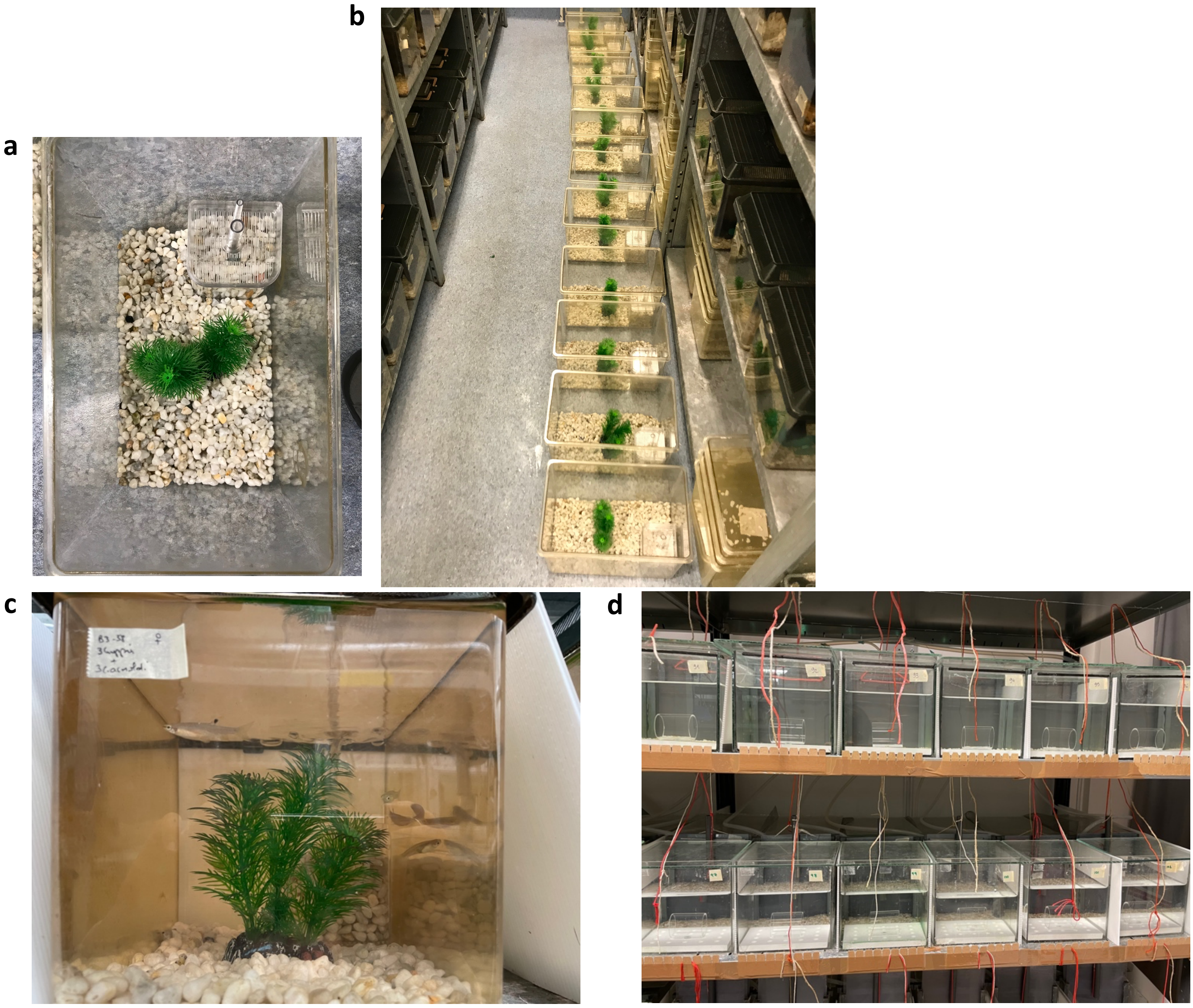

Figure S1. Photographs of the Experimental setup. (a) Preparation of a housing tank for social treatment. (b) Replicated housing tanks with identical physical enrichment: one plastic plant in the centre of the tank, an air filter in the right corner of the tank and 2 cm gravel. (c) an example of a housing tank containing the treatment of three guppies and three splash tetras. (d) experimental aquaria with test compartments prepared for the detour task with transparent cylinders.

BATCH 01	3 guppies	3 guppies + 3 tetra	6 guppies	3 guppies	3 guppies + 3 tetra	6 guppies	3 guppies	3 guppies + 3 tetra	6 guppies	3 guppies	3 guppies + 3 tetra	6 guppies	3 guppies	3 guppies + 3 tetra	6 guppies	3 guppies	3 guppies + 3 tetra	6 guppies	
tank_ID	B1-1	B1-2	B1-3	B1-4	B1-5	B1-6	B1-7	B1-8	B1-9	B1-10	B1-11	B1-12	B1-13	B1-14	B1-15	B1-16	B1-17	B1-18	total
baseline	1	1	1	1	1		1	1		1	1	1	0	0				1	11
test	2	2	5	2	2		2	2		2	2	5	3	3				5	37

BATCH02	3 guppies	3 guppies + 3 tetra	6 guppies	3 guppies	3 guppies + 3 tetra	6 guppies	3 guppies	3 guppies + 3 tetra	6 guppies	3 guppies	3 guppies + 3 tetra	6 guppies	3 guppies	3 guppies + 3 tetra	6 guppies	3 guppies	3 guppies + 3 tetra	6 guppies	
tank_ID	B2-19	B2-20	B2-21	B2-22	B2-23	B2-24	B2-25	B2-26	B2-27	B2-28	B2-29	B2-30	B2-31	B2-32	B2-33	B2-34	B2-35	B2-36	total
baseline	1	1		1	1	1	1	1	1	1	1	1	1	1	1	1	1	2	18
test	2	2		2	2	5	2	2	5	2	2	5	2	2	5	2	2	4	48

Figure S2. A schematic diagram showing the number of fish per tank and per treatment. The table shows the exact number of fish in every treatment tank, separated by batch identity. It indicated how many guppies we used to generate the brain morphology baseline dataset (those sampled before the cognitive tests) and for the test dataset (those sampled after performing in the inhibitory control, associative learning and reversal learning tests). Coloured in red are the six treatment tanks that got compromised and discarded throughout the experiment by initially having guppies looking like females but eventually developing secondary male traits (male colouration). The three social treatments are 3 guppies; 3 guppies + 3 tetra = three guppies and three splash tetras; and 6 guppies.

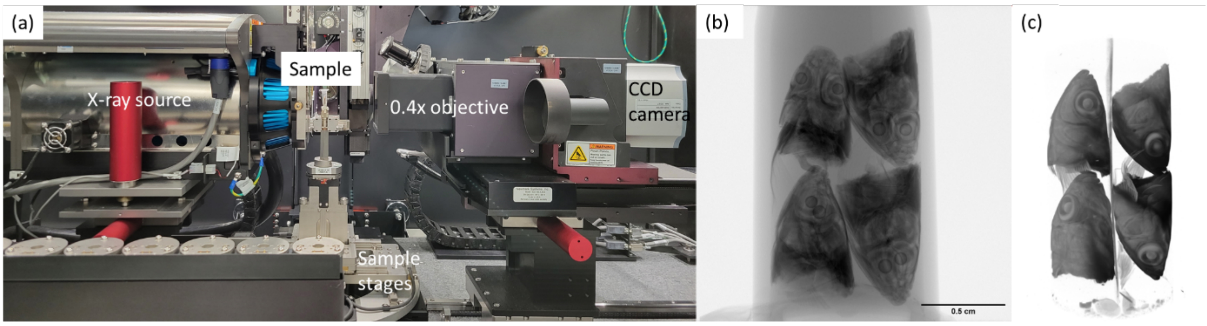

Figure S3. Brain scanning. (a) a photograph of the inside of the X-ray machine. (b) An example of a brain projection image. (c) 3D rendering of a micro-CT scan.

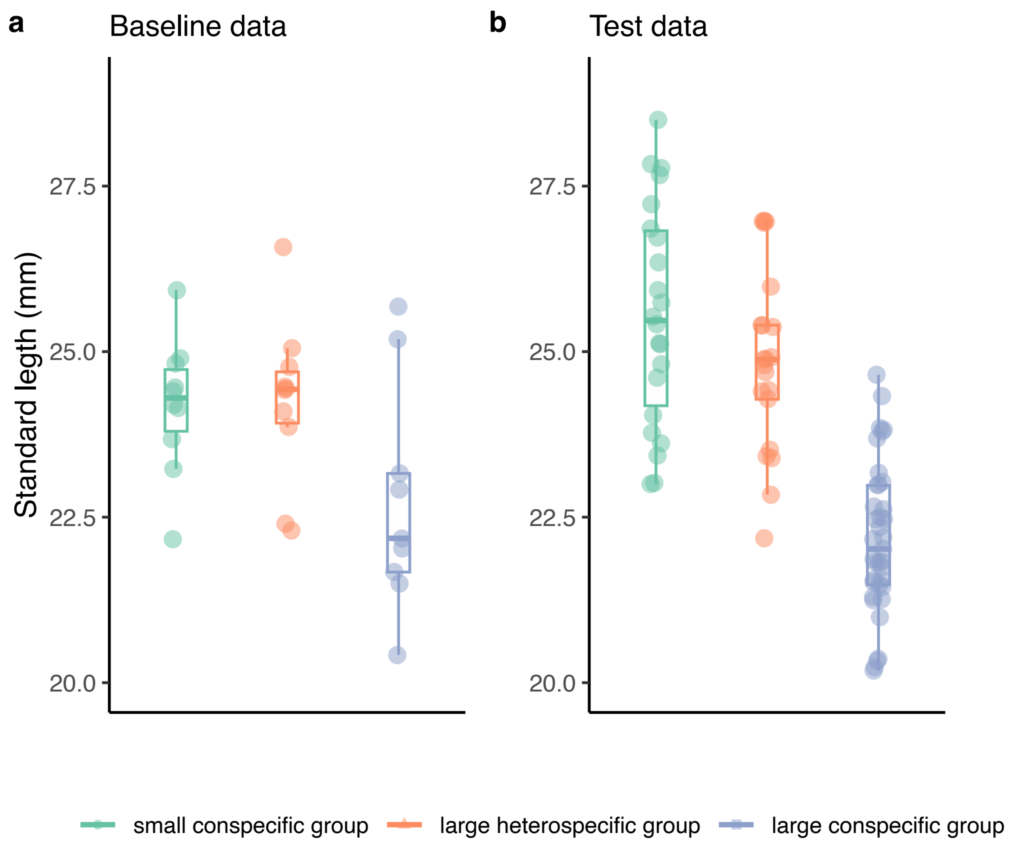

Figure S4. Female guppies' body standard length (mm) in the three social treatments. Boxplots of the median, interquartile range, and upper and lower quartiles, with raw data points. Guppies in the social treatment of six guppies are significantly smaller than the other guppies ($p \leq 0.05$).

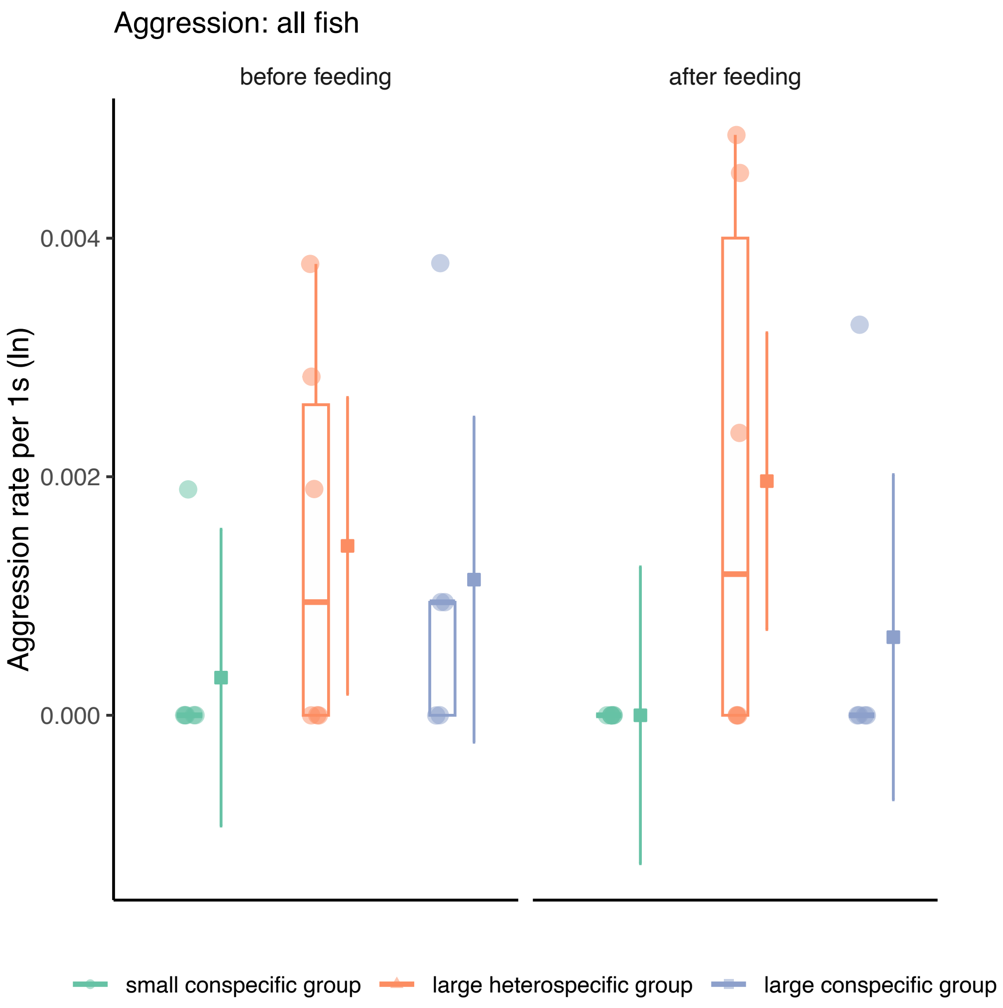

Figure S5. Aggression rates received by guppies in the holding tanks among all fishes (including splash tetras). The plots show estimates and 95% CI of model marginal effects, boxplots of median and interquartile of raw data, and the actual data points (N = 17) for aggression rates per 1 second of video recording before and after feeding as a function of social treatment. There was an effect of social treatment on these aggression rates ($p < 0.05$).

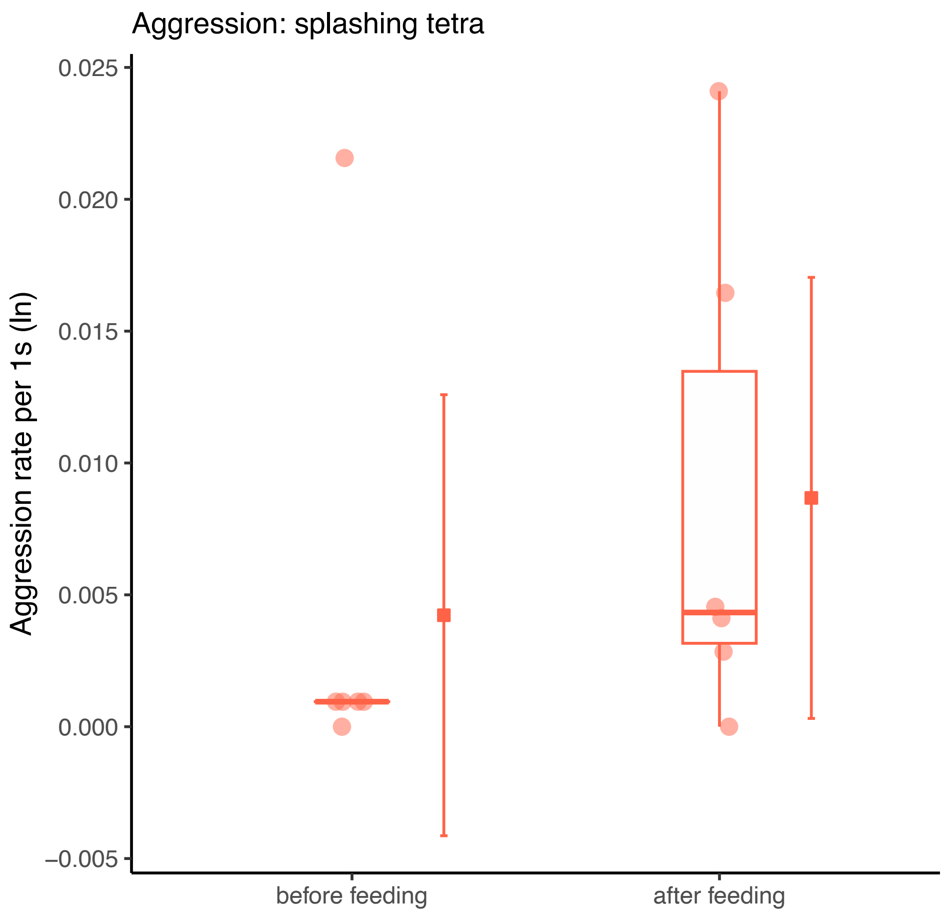

Figure S6. Aggression rates in the holding tanks among splash tetras in the three guppies and three splash tetras treatment. The plots show estimates and 95% CI of model marginal effects, boxplots of median and interquartile of raw data, and the actual data points (N = 6) for aggression rates per 1 second of video recording before and after feeding.

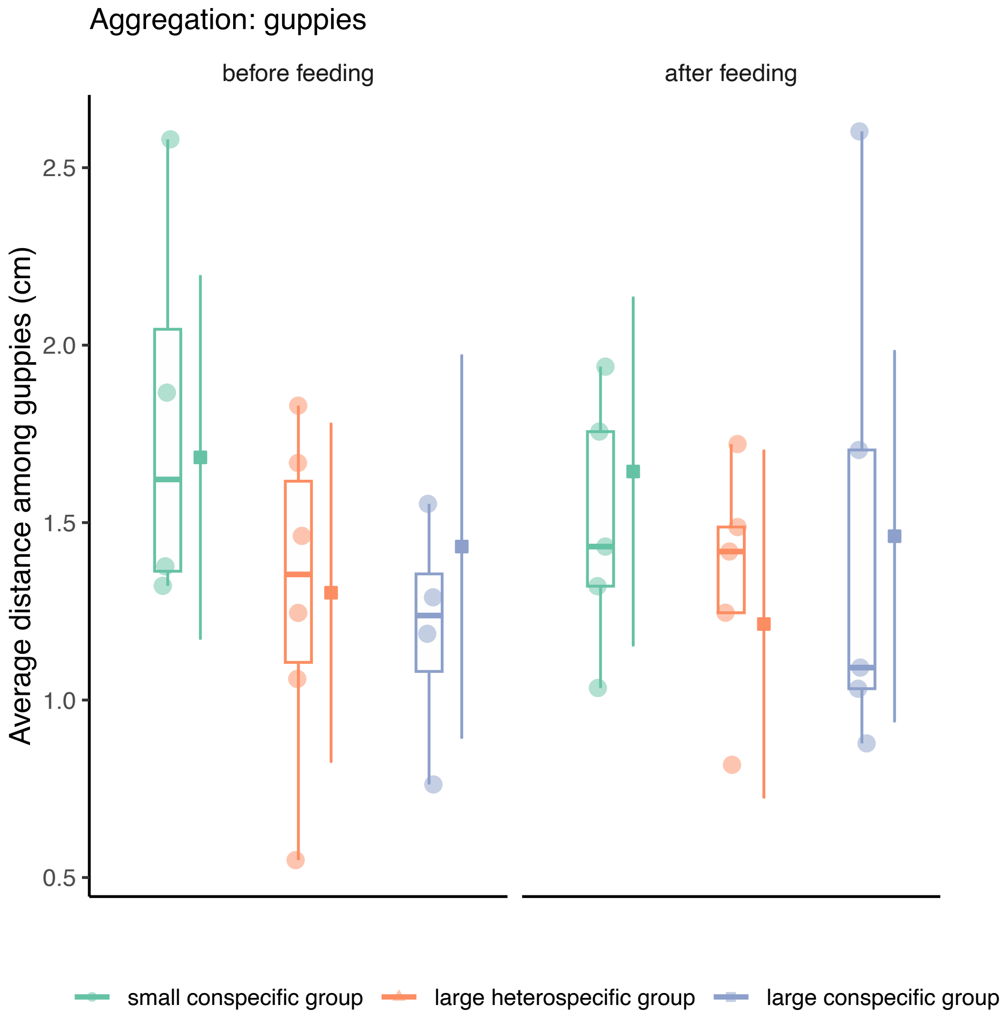

Figure S7. Aggregation distance among guppies. The plots show estimates and 95% CI of model marginal effects, boxplots of median and interquartile of raw data, and the actual data points (N = 17) for distance among guppies in cm, before and after feeding.

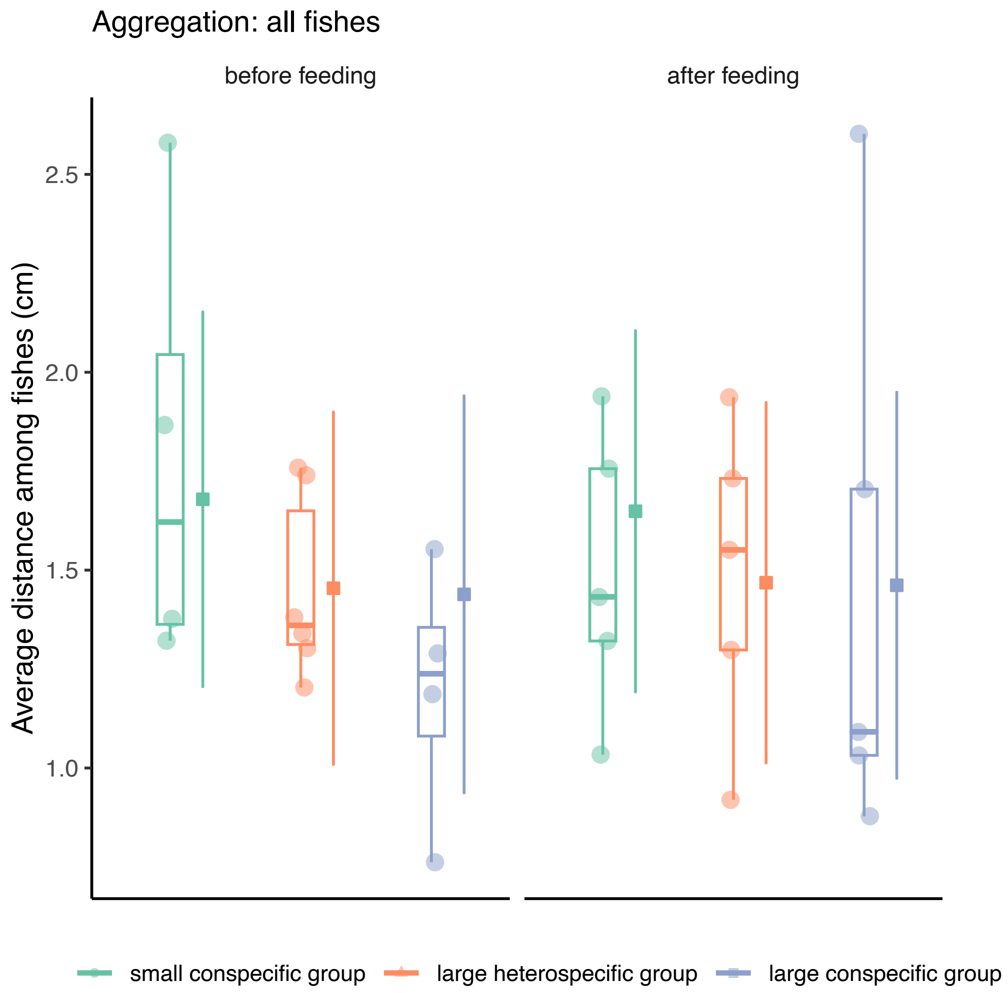

Figure S8. Aggregation distance among all fishes. The plots show estimates and 95% CI of model marginal effects, boxplots of median and interquartile of raw data, and the actual data points (N = 17) for distance among fishes in cm, before and after feeding.

Table S1. Summary of the statistical outcomes of the behavioural analyses. All models generating these statistics were Linear mixed effects models.

	aggression towards guppy		aggression among splash tetra		distance among guppies		distance among all fishes	
Predictor	Chisq	*p*	Chisq	*p*	Chisq	*p*	Chisq	*p*
treatment	**6.198**	**0.045**	–	–	1.709	0.426	0.650	0.723
feeding	0.015	0.902	1.250	0.264	0.159	0.690	0.004	0.947
social treatment x feeding	0.807	0.668	–	–	0.293	0.864	0.070	0.966
N	17		6		17		17	
Marginal-R^2 / conditional-R^2	0.179 / 0.203		0.063 / 0.448		0.088 / 0.838		0.035 / 0.850	
